# Prognostic Factors for Wilms Tumor Recurrence: A Review of the Literature

**DOI:** 10.3390/cancers13133142

**Published:** 2021-06-23

**Authors:** Alissa Groenendijk, Filippo Spreafico, Ronald R. de Krijger, Jarno Drost, Jesper Brok, Daniela Perotti, Harm van Tinteren, Rajkumar Venkatramani, Jan Godziński, Christian Rübe, James I. Geller, Norbert Graf, Marry M. van den Heuvel-Eibrink, Annelies M. C. Mavinkurve-Groothuis

**Affiliations:** 1 Princess Máxima Center for Pediatric Oncology, Heidelberglaan 25, 3584 CS Utrecht, The Netherlands; R.R.deKrijger-2@prinsesmaximacentrum.nl (R.R.d.K.); J.Drost@prinsesmaximacentrum.nl (J.D.); H.vanTinteren@prinsesmaximacentrum.nl (H.v.T.); m.m.vandenheuvel-eibrink@prinsesmaximacentrum.nl (M.M.v.d.H.-E.); A.M.C.Mavinkurve-Groothuis@prinsesmaximacentrum.nl (A.M.C.M.-G.); 2 Department of Medical Oncology and Hematology, Pediatric Oncology Unit, Fondazione IRCCS Istituto Nazionale dei Tumori, 20133 Milan, Italy; Filippo.Spreafico@istitutotumori.mi.it; 3 Department of Pathology, University Medical Center Utrecht, Heidelberglaan 100, 3584 CX Utrecht, The Netherlands; 4 Oncode Institute, 3584 CS Utrecht, The Netherlands; 5 Department of Pediatric Oncology and Hematology, Rigshospitalet, 2100 Copenhagen, Denmark; Jesper.Sune.Brok@regionh.dk; 6 Developmental Biology and Cancer Research and Teaching Department, University College London Great Ormond Street Institute of Child Health, London WC1N 1EH, UK; 7 Molecular Bases of Genetic Risk and Genetic Testing Unit, Department of Research, Fondazione IRCCS Istituto Nazionale dei Tumori, 20133 Milan, Italy; Daniela.Perotti@istitutotumori.mi.it; 8 Texas Children’s Hospital, Baylor College of Medicine, Houston, TX 77030, USA; Rajkumar.Venkatramani@bcm.edu; 9 Department of Pediatric Surgery, Marciniak Hospital, Fieldorfa 2, 54-049 Wroclaw, Poland; jgodzin@wp.pl; 10 Department of Pediatric Traumatology and Emergency Medicine, Wroclaw Medical University, Bujwida 44a, 50-345 Wroclaw, Poland; 11 Department of Radiation Oncology, Saarland University Medical Center and Saarland University Faculty of Medicine, D-66421 Homburg, Germany; Christian.Ruebe@uks.eu; 12 Division of Oncology, Cincinnati Children’s Hospital Medical Center, University of Cincinnati, Cincinnati, OH 45229, USA; James.Geller@cchmc.org; 13 Department of Pediatric Oncology and Hematology, Saarland University Medical Center and Saarland University Faculty of Medicine, D-66421 Homburg, Germany; Norbert.Graf@uks.eu

**Keywords:** Wilms tumor, recurrence, pediatric, prognosis

## Abstract

**Simple Summary:**

A Wilms tumor is a childhood kidney tumor. In high-income countries, 90% of patients with this tumor survive. However, the tumor recurs in 15% of patients. It is important to identify the patients at risk of recurrence in order to adjust treatment in such a way that recurrence may potentially be prevented. However, we are currently unable to determine precisely which patients are at risk of recurrence. Therefore, we present an overview of factors that influence the risk of recurrence, also known as prognostic factors. These factors range from patient-, tumor- and treatment-related characteristics to geographic and socioeconomic factors. In addition to these factors, biological markers, such as genetic alterations, should be studied more intensively as these markers may be able to better identify patients at risk of tumor recurrence.

**Abstract:**

In high-income countries, the overall survival of children with Wilms tumors (WT) is ~90%. However, overall, 15% of patients experience tumor recurrence. The adverse prognostic factors currently used for risk stratification (advanced stage, high risk histology, and combined loss of heterozygosity at 1p and 16q in chemotherapy-naïve WTs) are present in only one third of these cases, and the significance of these factors is prone to change with advancing knowledge and improved treatment regimens. Therefore, we present a comprehensive, updated overview of the published prognostic variables for WT recurrence, ranging from patient-, tumor- and treatment-related characteristics to geographic and socioeconomic factors. Improved first-line treatment regimens based on clinicopathological characteristics and advancing knowledge on copy number variations unveil the importance of further investigating the significance of biological markers for WT recurrence in international collaborations.

## 1. Introduction

In high-income countries, children with Wilms tumors (WT) have an overall survival (OS) rate of ~90% when adopting recent protocols of co-operative groups in pediatric oncology [1,2,3,4,5,6]. While OS rates are high, approximately 15% of all patients experience recurrent disease [7,8,9]. North American (Children’s Oncology Group Renal Tumor Committee (COG-RTC)), and European (International Society of Pediatric Oncology Renal Tumor Study Group (SIOP-RTSG)) study groups have developed different treatment approaches for children with renal tumors. Whereas the COG-RTC, building on the former trials from the National Wilms Tumor Study Group (NWTSG), opts for immediate nephrectomy in most cases of unilateral disease, the SIOP-RTSG treats children older than 6 months with pre-operative chemotherapy. The Associazione Italiana Ematologia Oncologia Pediatrica (AIEOP), Japan Wilms Tumor Study (JWiTS) and United Kingdom Children’s Cancer and Leukaemia Group (UKCCLG) in the past predominantly opted for upfront surgery-based approaches, but now adhere to SIOP protocols and participate in SIOP studies [1,3,4,5,10,11,12,13,14]. The factors currently used for upfront treatment stratification vary per treatment approach and are presented in Table 1. Although once proven to be of prognostic value in predicting tumor recurrence, the significance of these factors is subject to change with advancing knowledge and improved treatment regimens [15,16]. As a result, more sophisticated patient stratification criteria are required to optimize and personalize appropriate treatment and to enhance recurrence-free survival (RFS) rates. In this vein, a recent overview of prognostic variables for tumor recurrence in WT is lacking. This review aims to confirm the independent prognostic value of the currently applied variables and to explore novel factors that can predict risk of recurrence. For that purpose, the prognostic significance of patient, tumor and treatment characteristics were evaluated. Additionally, genetic aberrations, as well as geographic and socioeconomic factors (specifically in low-and lower-middle-income countries) that may influence the risk of recurrence were reviewed.

## 2. Materials and Methods

PubMed, Embase, Web of Science and the Cochrane library were searched for relevant articles. The search terms are presented in Appendix A. A total of 866 unique references were identified and, following an initial selection for relevance based on title and abstract, 291 full-text articles were screened (A.G., M.M.v.d.H.-E. and A.M.C.M.-G.). Articles were included if they met the following criteria: patients in the study were <18 years old; tumor recurrence was among the reported outcome measures; the study enrolled ≥100 WT patients; and a multivariable analysis was performed to identify independent prognostic factors. An expanded search (see Appendix A) and cross-reference checks identified another 59 unique and relevant references.

## 3. Worldwide WT Classification Systems

As a consequence of the difference in upfront treatment approach between the COG RTC and SIOP-RTSG, the co-operative groups have developed different tumor staging and histological classification systems (Table S1 (adapted from Phelps et al. and Fajardo et al. [17,18]), and Table S2). The staging and histological classification system of the COG is based on untreated tumor tissue. In contrast, SIOP staging and histological classification includes tumor response to pre-operative chemotherapy [2,19].

## 4. The Prognostic Value of Patient Characteristics

### 4.1. Age

Advanced age at diagnosis (as a linear factor and categorical factor (<24, 24–47 and ≥48 months)) is associated with higher tumor stage at diagnosis, higher frequency of high risk (HR) histology subtypes, especially diffuse anaplasia (DA), higher incidence of adverse loss of heterozygosity (LOH) patterns, and larger tumor volumes [20,21,22]. The largest study so far, performed on the SIOP 93-01 and SIOP 2001 cohorts including over 4500 WT cases by Hol et al., confirmed that age as a linear factor was significantly and independently associated with recurrence of disease [22]. Moreover, the predictive value of previously reported cutoff values of 6, 24 and 48 months, as well as 10 years of age, was validated in multivariable analysis. The prognostic value of age had previously been identified in a smaller subset of this cohort [23], and in previous studies in other large cohorts of SIOP and UKCCLG [20,24,25,26]. Interestingly, age was no longer significantly associated with recurrence when adjusted for gain of chromosome 1q in the SIOP 2001 and AIEOP (CNR-92 and TW2003) cohorts [27,28]. This is conceivably explained by the higher prevalence of 1q gain in older patients [29]. These results will be validated in the SIOP-RTSG UMBRELLA protocol [2,27,30]. During the NWTS-3, age at diagnosis in years (as a linear factor) was of prognostic significance for patients with stage II/III disease specifically [20]. For patients with stage I disease, the COG recognizes patients younger than 2 years old with favorable histology WT (FHWT), i.e., non-anaplastic tumors, as Very Low Risk Wilms Tumor (VLRWT) patients if tumor weight is below 550 g. These patients are treated with nephrectomy only, and do not receive radiotherapy or chemotherapy. The COG is now expanding this group based on LOH 11p15 status for patients up to 4 years of age and without taking tumor weight into account [31]. Dysregulated expression of IGF2 due to LOH 11p15 is associated with an increased risk of relapse in VLRWT patients, and may overcome the prognostic significance of age in this subset [32]. The association between LOH 11p15 and WT relapse will be further discussed in Section 7.2 Somatic mutations. JWiTS studied the prognostic value of age (<24 and ≥24 months) in 128 WT patients and did not confirm age to be an independent prognostic factor for recurrence [33].

### 4.2. Sex and Race

The prognostic significance of patient’s sex was studied in UKCCLG, COG and SIOP-initiated studies, all of which confirmed the lack of association between sex and tumor recurrence [20,22,23,25]. Similarly, race, which was studied only by the COG, was not found to be predictive of disease recurrence among patients treated during the NWTS-3 (comparing African-American patients to patients of different race) and AREN0532 (comparing Caucasian patients to patients of different race) [20,34].

## 5. The Prognostic Significance of Tumor Stage

Stage is one of the standard factors for primary treatment stratification. Patients with advanced-stage disease are treated with more intensive chemotherapy and additional radiotherapy (see Table S3 for current COG and SIOP radiotherapy guidelines) [35]. Since the current staging classification is based on easily available factors, it can also be used in low- and lower-middle-income countries [36]. That tumor stage is of prognostic value prior to start of therapy for primary WT is not up for debate here. However, the prognostic value of stage after first-line treatment is unsettled. Spreafico et al. have suggested that, due to the more sophisticated and tailored treatment regimens, disease outcome no longer varies much between patients of different local stages, which diminishes the independent prognostic value of the current staging system in predicting relapse after primary treatment [15].

### 5.1. Stage I and II

In the studies reported here, stage II patients generally received more intensive treatment than stage I patients, with the exception of anaplastic tumors in the UKW3 trial and FHWTs in the NWTS-5. Patients with stage I HR tumors in the SIOP-2001 study were treated with actinomycin-D, vincristine and doxorubicin (AVD), and patients with stage II intermediate risk (IR) tumors were randomized between AVD and treatment without doxorubicin (VA) [37]. Details on the studies reporting on relapse rates stratified by tumor stage are presented in Table 2, Table 3 and Table 4 (including stage III and IV). The 2-year RFS rates did not vary much between patients with non-anaplastic stage I and II WT in the NWTS-4 (92.5–94.9% vs. 85.9–89.7%) [38], and when adjusted for 1q gain in the NWTS-5, the event-free survival (EFS) rates remained comparable between stage I and II (Table 2) [29]. Additionally, in UK and AIEOP studies, patients with stage I and II disease presented with comparable RFS and EFS rates (Table 3) [5,25]. In a combined multivariable analysis of WT patients registered on the SIOP 93-01 and subsequent 2001 studies, EFS was significantly better for stage I compared to stage III, but not when compared to stage II (Table 4) [22]. Irtan et al. observed that, among patients with stage I–IV WT, children with local stage II disease were significantly more likely to suffer from distant recurrence than patients with local stage I in the SIOP 2001 cohort [23].

Tumors that extend beyond the kidney are considered at least stage II in both the SIOP and COG staging system (tumors were considered stage III in case of atrial extension (COG) and residual intravascular extension or piecemeal resection (COG and SIOP)). In neither SIOP (SIOP-1 and SIOP-2) nor COG (NWTS-3) studies was capsular penetration found to be a prognostic factor for recurrence, probably because this was already accounted for by adjusting treatment [20,39]. Similarly, no significant difference in RFS between patients with and without intrarenal vascular invasion (NWTS-3) or intravascular extension (combined analysis of intracaval (at least stage II) and atrial (stage III) tumor extension) (NWTS-4) was observed when adjusted for stage and histology [20,40]. Further, the UKW3 trial reported no difference in EFS for patients with and without intracaval or atrial tumor extension [41]. Although information on treating centers could not be deduced from the referenced literature, it is conceivable that patients with intracaval and atrial tumor extension were treated by experienced surgeons in leading centers, resulting in excellent EFS rates. In addition, patients received intensified treatment with increasing stage. However, from the NWTS-5 onwards, stage I and II FHWT COG patients have received the same therapy, resulting in comparable relapse rates [29,42]. This suggests that treatment stratification by LOH 1p/16q and 1q status overcomes the prognostic significance of capsular penetration and intravascular tumor extension in these stage II patients.

**Table 2:** Prognostic significance of stage according to NWTS/COG studies.

Authors	Ref.	Study	*n*	Tumor Characteristics and Stratifiers	Survival Rates	*p*-Value ^1^
D.M. Green et al. 1998	[38]	NWTS-4	1543	Stage I–IV, FH Pulse-intensive (single-dose)/standard (divided-dose) chemotherapy regimen	2-year RFS: pulse-intensive/standard regimen Stage I: 94.9/92.5% Stage II: 85.9/89.7% Stage III: 91.1/95.3% Stage IV: 80.6/81.3%	NA
E.J. Gratias et al. 2016	[29]	NWTS-5	1114	Stage I–IV, FH 1q gain/no 1q gain	8-year EFS: 1q gain/no 1q gain Stage I: 85% (72–98%)/95% (91–99%) Stage II: 81% (71–91%)/87% (83–92%) Stage III: 79% (70–87%)/89% (84–94%) Stage IV: 64% (48–79%)/91% (83–99%)	NA
D. B. Dix et al. 2019	[42]	NWTS-5 AREN0532 AREN0533	168	Stage I–IV, FH, with LOH 1p/16q Study protocol: NWTS-5/AREN	4-year EFS: NWTS-5/AREN study protocols Stage I–II: 68.8% (55.2–82.3%)/87.3% (75.1–99.5%) Stage III–IV: 61.3% (44.9–77.6%)/90.2% (81.7–98.6%)	NA
N.C. Daw et al. 2020	[43]	AREN0321	66	Stage II–IV, DA	4-year EFS: Stage II: 86% (68.8–100%) Stage III: 80.9% (65.8–96%) Stage IV: 41.7% (19.6–63.7%)	NA
J.S. Dome et al. 2006	[44]	NWTS-5	124	Stage I–IV, AH	4-year EFS Stage I: 69.5% (46.9–84.0) Stage II: 82.1% (62.3–92.1%) Stage III: 68.3% (53.6–79.3%) Stage IV: 37.5% (15.4–59.8%)	NA
D.M. Green et al. 1994	[45]	NWTS-3 NWTS-4	59	Stage II–IV, DA Treatment without Cy/with Cy	4-year RFS: without Cy/with Cy Stage II: 40.0%/71.6% Stage III: 33.3%/58.7% Stage IV: 0.0%/16.7%	NA

^1^*p*-values for comparisons of outcome between stage were not calculated/reported. Abbreviations: AH, anaplastic histology (focal and diffuse anaplasia); Cy, cyclophosphamide; DA, diffuse anaplasia; Dox, doxorubicin; EFS, event-free survival; FH, favorable histology; LOH, loss of heterozygosity; NA, not applicable; *n*, number of patients; Ref., reference.

**Table 3:** Prognostic significance of stage analyzed in MVA in UKCCLG, AIEOP and JWiTS studies (>100 patients).

Authors	Ref.	Cooperative Group	Study	*n*	Outcome	Comparison	Hazard Ratio	*p*-Value
T. Koshinaga et al. 2018	[4]	JWiTS	JWiTS-2	178	EFS	I–III vs. IV ^1^	Significant (Log-rank)	<0.01
F. Spreafico et al. 2017	[5]	AIEOP	TW2003	453	EFS	I vs. II	1.13 (0.56–2.29)	n.s.
						I vs. III	0.96 (0.40–2.30)	
						I vs. IV	1.90 (0.82–4.42)	
S. Irtan et al. 2015	[25]	UKCCLG	UKW3	635	Local RFS	I vs. II I vs. III I vs. IV	n.s. in UVA	n.s.
					Distant RFS	I vs. II I vs. III I vs. IV	0.99 (0.46–2.12) 1.46 (0.80–2.27) 2.35 (1.27–4.33) In UVA	n.s. in MVA
M. Haruta et al. 2019	[33]	JWiTS	1987–2015	128	EFS	I–III vs. IV ^1^	0.86 (0.79–0.93) vs. 0.54 (0.25–0.82)	0.006
B. Messahel et al. 2009	[46]	UKCCLG	UKW1-3	452	RFS	I–II vs. III–IV ^2^	1.35 (0.78–2.31)	0.28
H. Segers et al. 2013	[47]	SIOP/UKCCLG	SIOP2001, UKW2-3 ^3^	272	EFS	I vs. II	1.23 (0.49–3.12)	0.66
						I vs. III	2.29 (1.01–5.21)	0.05
						I vs. IV	3.63 (1.62–8.13)	0.002

^1^ Univariable analysis only. ^2^ Stage I patients comprised 66% of stage I/II population, and stage III patients comprised 65% of stage III/IV population. ^3^ Unadjusted for treatment approach (immediate surgery (UKW2 and half of UKW3) or pre-operative chemotherapy (half of UKW3 and SIOP 2001)). Abbreviations: EFS, event-free survival; MVA, multivariable analysis; *n*, number of patients; n.s., not significant; Ref., reference; RFS, relapse-free survival; UVA, univariable analysis.

**Table 4:** Prognostic significance of stage analyzed in MVA in UKCCLG, AIEOP and JWiTS studies (>100 patients).

Authors	Ref.	Study	*n*	Outcome	Comparison	Hazard Ratio	*p*-Value
J.A. et al. Hol 2019	[22]	SIOP9301, SIOP2001	4596	I vs. II	EFS	1.13 (0.91–1.41)	0.28
				I vs. III		1.60 (1.28–2.00)	<0.0001
				I vs. IV		2.97 (2.40–3.67)	<0.0001
S. Irtan et al. 2019	[23]	SIOP2001	2971	I vs. II Abdominal stage	Local RFS	n.s.	n.s.
					Distant RFS	1.58 (1.15–2.17)	0.005
				I vs. III Abdominal stage	Local RFS	n.s.	n.s.
					Distant RFS	1.52 (1.00–2.32)	0.05
				I–III vs. IV	Local RFS	n.s.	n.s.
					Distant RFS	2.50 (1.88–3.31)	<0.0001
A. Weirich et al. 2004	[24]	SIOP-9/GPOH	440	I–II vs. III	RFS	n.s.	n.s.
				I–III vs. IV–V ^1^		2.7	0.0003
T. Chagtai et al. 2016	[27]	SIOP2001	585	I vs. II	EFS	1.27 (0.71–2.27)	0.43
				I vs. III		1.52 (0.83–2.79)	0.17

^1^ Three out of 59 stage IV–V patients were stage V. Abbreviations: EFS, event-free survival; MVA, multivariable analysis; *n*, number of patients; n.s., not significant; Ref., reference; RFS, relapse-free survival; UVA, univariable analysis.

### 5.2. Stage III

The criteria for stage III are different for COG and SIOP, and are listed in Table S1. It may be noted that the COG studies treated all patients with tumor spill (including tumor biopsy) as stage III patients, whereas only patients with diffuse tumor spill (i.e., beyond the flank) were considered stage III during the NWTS-3, 4 and 5. However, tumor biopsy or local spill are not commonly the only cause for stage III classification and the difference in staging criteria therefore impacts very few patients [34]. The 2-year RFS reported for stage III FHWT patients registered in the NWTS-4 (91.1–95.3%) was comparable to stage I and II patients [38]. When adjusted for LOH 1p/16q (4-year RFS) and 1q gain (8-year RFS) in the subsequent NWTS-5 and AREN0532 and AREN0533 studies, relapse rates remained similar in all FHWT non-metastatic stages (Table 2) [29,42]. Similarly, in two UK studies with overlapping cohorts (UKW1-3 [46], and UKW2–3 and SIOP 2001 [47]), stage III was no longer of prognostic significance for recurrence when adjusted for copy number variations, including LOH 1p and/or 16q in both studies and 1q gain only in the latter (Table 3). Unadjusted for copy number variations, but adjusted for anaplasia (Focal anaplasia (FA) and DA combined), AIEOP did not observe an association between stage I, II or III and recurrence in the TW2003 study [5]. In univariable analysis, JWiTS reported on RFS of >90% for all patients with local stage tumors registered on JWiTS-2 [4]. These rates had significantly improved when compared to the first JWiTS study (5-year RFS of 82.0% (unadjusted analysis)) [13]. EFS of patients with stage I–II tumors compared to stage III tumors was not significantly different in the SIOP-9/GPOH (Gesellschaft für pädiatrische Onkologie und Hämatologie) (Table 4) [24]. Analysis of non-anaplastic WTs in the SIOP 93-01 study, however, revealed that patients with stage III disease had significantly inferior EFS than stage II patients when adjusted for (blastemal) histology [48]. Stage III patients appeared to have an increased risk of recurrence compared to stage I patients in the combined analysis of the SIOP 93-01 and 2001 studies [22]. This difference was no longer significant when adjusted for 1q gain, or when stratified by location of recurrence in the SIOP 2001 cohort [23,27].

#### 5.2.1. Lymph Node Status

Invasion of abdominal lymph nodes, including the renal hilum and periaortic chain, which classifies patients as stage III, appears to be a prominent risk factor for recurrence in COG patients with upfront stage III disease [34,49,50]. In the NWTS-5 trial, the combined presence of positive lymph nodes and microscopic residual disease was predictive of recurrence based on multivariable analysis [50], and the AREN0532 study demonstrated that the combination of LOH of 1p or 16q in addition to lymph node positivity was associated with poorer EFS (based on log rank analysis) in patients with stage III FHWT tumors [34]. In patients treated by AIEOP, lymph node involvement was the only reason for stage III disease that was significantly associated with disease-free survival in multivariable analysis [14].

SIOP also considers invasion of lymph nodes as a criterion for stage III classification, including the presence of viable tumor cells, chemotherapy-induced changes or necrosis in lymph nodes [19]. In the SIOP 93-01 and 2001 study, lymph node positivity was not associated with lower EFS when compared to stage III patients without lymph node involvement [23,49]. This was also observed in an unadjusted analysis of the UKW3 [51]. The lack of association between recurrence and lymph node positivity in the context of pre-treated tumors may be caused by the fact that patients with different histological characteristics at involved lymph nodes (i.e., only chemotherapy induced changes or necrosis versus viable tumor), which may be related to a different prognosis, all receive doxorubicin and RT.

Omission of lymph node sampling likely results in understaging and subsequent undertreatment of patients with occult positive lymph nodes. Patients who had no lymph nodes sampled had worse EFS than those with positive lymph nodes during the NWTS-4 [52]. Kieran et al. reported that the chance of finding a positive lymph node was higher when at least seven lymph nodes were sampled, and hence, the UMBRELLA protocol now advises to sample at least seven lymph nodes [2,53]. Adherence to lymph node sampling protocols, however, requires improvement, as became apparent from the SIOP 2001 and UK 2013 IMPORT registries [54]. Historically, the number of LNs sampled during nephrectomy for WT was left to the discretion of the NWTS/COG operating surgeon [53]. However, the COG will now require sampling of at least one lymph node for enrolment onto future studies.

#### 5.2.2. Tumor Spill

From the NWTS-3 onwards, intra-operative tumor spill had been defined by the NWTSG as either local (stage II), when spill was confined to the flank, or diffuse (stage III) when the entire peritoneal cavity was contaminated. Patients with any type of intra-operative spill during surgery in the NWTS-4 study had an almost three-fold increased risk of recurrence [52]. Closer analysis revealed that primarily stage II patients were at risk of recurrence after surgical tumor spill, which was supported by a subsequent NWTS-4 analysis [55]. Consequently, trials initiated by the COG treated all primary nephrectomy patients who experience tumor spill (including tumor biopsy) as having stage III tumors, prescribing radiotherapy as part of adjuvant therapy for patients with unilateral disease. The use of radiotherapy appears to prevent tumor recurrence in case of spill, as was observed in stage III patients in the NWTS-4 and 5 studies [50,52]. Gow et al. demonstrated that 12.6% of stage III patients in the AREN03B2 study population were stage III based on tumor spill alone, and that risk of surgical spill increased with increasing tumor size [56]. Use of pre-operative chemotherapy has been shown to result in tumor size reduction [57], thereby reducing the risk of intra-operative spill and the associated risk of tumor recurrence. As proposed by Gow et al., some COG patients may thus have avoided spill and potentially also tumor recurrence if they had been treated with pre-operative chemotherapy. Such patients would, however, still require doxorubicin and flank RT according to the COG treatment protocol [56].

AIEOP, reporting low numbers of cases (*n* = 56, 12%), did not identify an association between intra-operative tumor spill (which was not a criterion for upstaging to stage III if the spill was local) and RFS in multivariable analysis [5]. In the UKW3 study, patients with local tumor spill were considered stage II patients and treated without RT, yet, unlike reported by the NWTSG, tumor spill was not associated with disease recurrence upon multivariable analysis (timing of spill, pre-operatively or intra-operatively, was not reported) [25]. SIOP delineates between major tumor spill (spread throughout the (retro)peritoneal cavity visible on imaging or during surgery) and minor tumor spill. Both major and minor spill can occur before or during surgery, and both used to be criteria for upstaging to stage III disease. All patients with spill were thus treated with radiotherapy (whole abdominal radiotherapy in case of major spill). The UMBRELLA protocol now only classifies tumors as stage III in case of spill if viable tumor is seen microscopically at the rupture site of the nephrectomy specimen. If this is not the case, the tumor is staged according to other pathological criteria [2,30]. In the SIOP-9, SIOP 93-01 and SIOP 2001, EFS was not affected by tumor spill (comparing any spill (major/minor and pre-operative/intra-operative spill) to no spill. There was no significant difference in EFS when comparing major and minor spill in the SIOP-9 and SIOP 93-01 cohorts [23,58].

#### 5.2.3. Tumor Biopsy

In both COG and SIOP protocols, diagnostic tumor biopsy is not standard of care, but may be performed in case of atypical presentation of the renal tumor, unusual biological findings, and unusual findings by imaging. Whereas the NWTS did not upstage tumors that had undergone tumor biopsy to stage III, the COG does, regardless of the type of biopsy (open, tru-cut or fine needle aspiration) [34,55]. The SIOP, UKCCLG and AIEOP groups do not upstage tumors that have undergone a needle biopsy to stage III [2,5,25]. Among UK patients who were randomly assigned pre-operative percutaneous core/fine needle biopsy, 1 out of 182 patients experienced a needle track recurrence, and biopsy was not associated with recurrence in multivariable analysis [25,59]. Similarly, needle biopsy was not associated with recurrence in multivariable analyses performed with patients who were treated with pre-operative chemotherapy during the SIOP 2001 study [23], nor with patients who underwent immediate nephrectomy in accordance with the AIEOP TW2003 study protocol [5].

#### 5.2.4. Residual Disease

In multivariable analysis, microscopic residual disease was prognostic for stage III FH patients in the NWTS-5 [50]. In the AREN0532, gross residual disease was not associated with EFS among stage III FHWT based on log rank analysis. The prognostic significance of microscopic residual disease could not be assessed in this study [34]. The SIOP 2001 and UKW3 studies did not identify a significant association between positive resection margins and recurrence or EFS in multivariable analysis [23,25].

#### 5.2.5. Summary

None of the criteria for stage III disease described above (positive lymph node status, tumor spill, biopsy and residual disease) appear to be associated with tumor recurrence in patients treated according to SIOP RTSG strategies [23,24,27,47,49,58]. COG patients with LOH 1p or 16q and positive lymph nodes have the highest rate of relapse among stage III patients, despite use of radiotherapy and three-drug chemotherapy [34,49,50]. This indicates an important role for biological markers in treatment stratification and stresses the importance of further unraveling their prognostic significance.

### 5.3. Stage IV

The NWTS-5 study did not observe an increased relapse rate among stage IV FHWT, compared to localized FHWT, stratified by 1q gain [29]. However, inferior EFS was observed in case of stage IV anaplastic histology WT (AHWT) (Table 2) [43,44,45]. AIEOP TW2003 and UKW3 reported similar findings: EFS was not different between stage I and stage IV patients when adjusted for tumor histology [5,25] (Table 3). Stage IV JWiTS patients experienced inferior EFS when compared to patients with local stage disease, but analyses were unadjusted for other clinicopathological factors [4,33]. When comparing outcome of patients with local stage tumors registered on the SIOP studies (SIOP-9 and SIOP 2001) to patients with metastatic disease, stage IV patients experienced inferior RFS (Table 4) [23,24]. The SIOP 93-01 study showed that especially stage IV patients with local stage III disease were at risk of tumor recurrence [60]. Verschuur et al. identified HR histology and higher blastemal residual volume in the primary tumor (applying a cut-off of 10 mL) as the only independent prognostic factors for EFS in case of stage IV disease treated by SIOP-RTSG protocols [61]. In patients with stage IV HR histology, 5-year EFS significantly improved in patients treated with radiotherapy to metastatic sites [62].

Most patients with stage IV disease present with pulmonary metastases, although the location of metastases (pulmonary, hepatic or other) at diagnosis of FHWT does not bear prognostic significance according to a NWTSG study (log rank analysis) [63]. Overall, the response of metastatic disease to chemotherapy is currently believed to be a more important prognostic indicator than the presence of metastases. The prognostic significance of response to therapy of metastatic disease has been shown in stage IV patients with lung metastases only, differentiating between rapid complete responders (who have a significantly better prognosis) and slow/incomplete responders [60,64]. In addition, data from the SIOP 93-01 and 2001/GPOH study showed that lung irradiation could be avoided if complete remission was achieved through pulmonary metastasectomy, demonstrating once more that response to treatment is of prognostic evidence [65].

Pulmonary lesions that are identified only by computed tomography (CT) because of the small dimension of the nodules (so called “CT-only nodules”, defined as having a concurrent normal chest x-ray) may also have a different prognostic value when compared to overt metastases. Patients with CT-only nodules were not considered stage IV by the NWTSG, yet they appeared to have a better 5-year EFS when they were treated with additional doxorubicin in NWTS-4 and 5. Whole lung irradiation, however, had no added value in patients with CT-only nodules [66]. The COG now classifies WT with CT-only nodules as stage IV disease, and patients are treated as such, eligible for omission of radiotherapy pending biology and response to chemotherapy. SIOP 2001 reported a 3-year EFS of 74% (95% CI: 65–84%) in case of CT-only nodules, which was significantly inferior compared to patients with true localized disease (87% (95% CI: 86–89%)). Unlike the COG findings, in SIOP 2001, EFS was similar whether CT-only nodules were treated as local disease or as stage IV, but therapy often included doxorubicin. The independent role of whole lung irradiation in SIOP CT-only patients was not investigated [67]. CT-only nodules with diameter of ≥3 mm will be treated as metastases in the UMBRELLA protocol [2].

### 5.4. Bilateral Disease (Stage V)

Currently, whenever feasible, both COG and SIOP protocols advise pre-operative chemotherapy followed by either nephron-sparing surgery (NSS) or radical nephrectomy in case of stage V WT [1]. Whereas the COG advocates initial treatment with VAD, or with vincristine, actinomycin-D, doxorubicin, cyclophosphamide, carboplatin and etoposide in case of DA histology (based on tumor biopsy) [68], SIOP treats stage V patients initially with 6 weeks of VA, potentially followed by additional VA or etoposide/carboplatin treatment prior to surgery, dependent on the response to initial treatment [2]. In an unadjusted analysis AIEOP reported a 4-year disease-free survival rate of 51% (95% CI: 38–64%) for patients with DA and post-chemotherapy blastemal histology tumors, and an EFS of 72% (95% CI: 69–75%) for patients without these histological features [69]. In this study, treatment consisted of initial VA, or VAD in case of metastatic disease or signs of venous tumor thrombi. The COG similarly reported that anaplasia was associated with inferior EFS rates in stage V patients [44,68,70]. COG AREN0534 obtained an unadjusted 4-year EFS rates of 84.2% (95% CI: 75.2–93.1%) for patients without anaplasia (no FA or DA), and 58.2% (95% CI: 15.7–100%) for patients (*n* = 17) with DA in at least one kidney. Blastemal type histology after 6 weeks of pre-operative chemotherapy (without FA or DA), however, was associated with an EFS of 81.8% (95% CI: 42.3–100%) (*n* = 11), and it remains a curiosity that these patients had a favorable outcome [68]. UWK3 estimated a 2-year EFS of 70.2% (95% CI: 55.0–81.1%) in case of non-anaplastic WT (treated pre-operatively with VAD) [3]. JWiTS, who evaluated outcome of 31 non-anaplastic bilateral WT cases registered in their database from 1996 to 2011, observed a 10-year EFS of 85.5% [71]. Patients treated by JWiTS underwent initial tumor biopsy or resection and subsequently received chemotherapy according to the abdominal stage and tumor histology. SIOP reported on an estimated 5-year EFS of 83.4% and 80% for all stage V patients treated respectively in France during the SIOP 93-01, and in Germany during the SIOP-9, but the number of patients with DA in both studies was too low (0–4%) to draw conclusions on the impact of histological type in patients with bilateral WTs [24,72]. A combined analysis of SIOP/GPOH-9, 93-01 and 2001 identified metastatic disease and an underlying syndrome or malformation as independent prognostic factors for disease progression in stage V patients, and anaplasia (FA or DA) (*n* = 14/120) was not associated with progression of disease (*n* = 5/14) [73].

Altogether, with more intensive, standardized chemotherapy and enhanced guidelines on surgery in stage V WT, EFS rates of patients with non-anaplastic bilateral disease appear to slowly approach those of unilateral WT. However, with the exception of the GPOH analysis, EFS rates were unadjusted for potential underlying genetic risk in case of syndromic WT, which is often associated with nephroblastomatosis that can progress to WT and lead to bilateral disease [73,74].

## 6. The Prognostic Significance of Tumor Histology

The two WT histological classification systems, currently in use by the cooperative groups, are those of COG and SIOP (Table S2) [30,35], reflecting their diverse attitude towards pre-operative chemotherapy and immediate nephrectomy. The prognostic significance of anaplastic histology, blastemal-type after pre-operative chemotherapy in the unilateral setting, and other SIOP histological subtypes will be further discussed.

### 6.1. Anaplasia

Anaplasia, currently diagnosed based on tumor cell morphology, is found in approximately 5 to 10% of WTs. In the first NWTS study, Beckwith et al. already demonstrated the association of anaplasia and adverse outcome when compared to non-anaplastic histology [75]. Subsequent NWTS studies additionally reported a significant difference in RFS and OS between FA and DA tumors [76]. The 4-year EFS for patients with FA in the NWTS-5 was 74.9% (95% CI: 59.9–85.0%), and 54.9% (95% CI: 46.2–62.7%) for patients with DA [44]. In a SIOP case series of 86 anaplastic tumors, 4-year disease-free survival was 69% in patients with FA, and 37% in patients with DA [77]. The presence of anaplasia (both FA and DA) is associated with older age (rare under the age of two), and DA tumors are more often found in high-stage tumors (III–IV) [44,77]. However, even when adjusted for age and stage, FA and DA in UKCCLG patients, and DA in SIOP patients remained independent prognostic factors for recurrence [22,25]. Additionally, in stage V disease, DA histology significantly affected EFS, as reported by COG and AIEOP [68,69]. It is noteworthy that anaplasia is thus regarded an adverse prognostic factor regardless of initially received treatment.

Anaplasia has previously been associated with *MYCN* mutations [78], and *TP53* mutations [79,80]. SIOP-RTSG and COG RTC investigators jointly studied the *TP53* mutational status in 40 DA WT samples. Estimates of 5-year EFS rates were 80% in patient with wild type TP53 and 44% for patients with mutated *TP53*. The corresponding hazard ratio for an event was 3.89 (95% CI: 1.26–16.9) for patients with mutated *TP53* [81]. This study was unable to interrogate the potential confounding effect of stage, unlike the study by Ooms et al., who stratified 118 patients with DA by stage. Upon log-rank analysis, Ooms et al. concluded that specifically patients with high-stage tumors (stage III–IV) and mutated *TP53* have an increased risk of recurrence [82]. Wegert et al., who studied the prevalence of *TP53* mutations in fatal WT cases irrespective of tumor histology, reported that mutated *TP53* was closely associated with anaplasia, although similar mutation rates were found in non-fatal anaplastic cases. Mutated *TP53* was also observed in non-anaplastic fatal cases, including 7/17 blastemal-type tumors, suggesting that the molecular diagnosis of *TP53* mutations may detect additional high-risk WT cases. Future studies should explore the prognostic significance of *TP53* mutations for recurrence in apparent histology-based non-anaplastic WT.

### 6.2. Blastemal-Type WT

Blastemal-type WT, defined as tumor comprised of at least ≥66% viable blastema after pre-operative chemotherapy [30], can, by definition, only be classified in tumors treated with chemotherapy prior to surgery [83,84]. Blastemal-type WT must not be confused with pre-chemotherapy blastemal histology, which is not associated with increased risk of recurrence or poor survival [85]. In fact, blastemal cells are very responsive to chemotherapy, and it is the residual blastemal component that is therapy resistant and pertains to the increased risk of recurrent disease in blastemal-type WT that will be discussed here [19,86]. RFS was significantly lower in blastemal-type tumors (82% 5-year EFS in 93-01) when compared to other non-anaplastic histological subtypes (93–100% EFS) that are recognized by SIOP [6,86]. Van den Heuvel-Eibrink et al. reported on a 5-year EFS of 80% (95% CI: 75–86%) in SIOP 2001, and 67% (95% CI: 59–76%) in SIOP 93-01 for localized blastemal WT [83]. COG AREN0532 reported on 116 patients with stage III FHWT who had received chemotherapy prior to nephrectomy. Seven of these patients presented with blastemal-type tumors (according to SIOP classification), five of which relapsed. This resulted in a 4-year EFS of 28.6% (95% CI: 0–75.9%), which was significantly lower than EFS for low- and intermediate-risk COG patients [34]. To further specify the definition of the blastemal-type risk group, SIOP UMBRELLA is currently studying the significance of stratifying patients based on remaining absolute blastemal volume, rather than relative proportional volume [2]. Absolute blastemal volume was identified as an independent prognostic factor for EFS, and retrospective preliminary data suggests that an absolute blastemal volume of >20–50 mL might serve as a useful biomarker for adverse prognosis [87]. Patients with blastemal type WT treated for bilateral disease on study AREN0534 had a favorable outcome [68]. Whether blastemal type portends a different prognosis in the bilateral setting requires further investigation.

### 6.3. SIOP Non-Anaplastic and Non-Blastemal Histological WT Subtypes

Since none of the patients with completely necrotic tumors in two studies relapsed, and also OS was excellent (5-year OS in SIOP-9: 98% (95% CI: 91–100%) and 6.5-year OS in SIOP-9/GPOH: 96% (95% CI: 88.3–100%), these tumors are considered low-risk (LR) tumors [19,24,86,88]. The only two reported deaths in these studies were therapy-related in one patient and due to veno-occlusive disease unrelated to WT in the other [24,88]. The other (non-anaplastic and non-blastemal) histological types, including regressive, mixed, stromal and epithelial WT, are treated as IR tumors. Weirich et al. observed that completely necrotic, stromal and epithelial WTs had a significantly better RFS than regressive, mixed-type or blastemal WT [24,86]. These findings were supported by results from the total SIOP 93-01 cohort [89]. As expected from the association between histological type and RFS, risk groups represent independent prognostic factors for recurrence [22,23,27,57].

### 6.4. Tumor Volume

Change in tumor volume, another measure of tumor response to chemotherapy, is associated with histological risk group [6]. In the SIOP 93-01 and 2001 cohorts, tumor volume at surgery was a prognostic factor for recurrence, independent of histological subtypes or risk groups [22,23]. The change in tumor volume appeared more prominent in completely necrotic WT than in viable tumors [88], and volume reduction after pre-operative chemotherapy was less pronounced in stromal and epithelial tumors compared to regressive, mixed and blastemal tumors [89]. In a post hoc analysis of stage II/III IR non-stromal and non-epithelial WTs in the SIOP 2001 trial, significantly more relapses occurred in patients with a tumor volume of ≥500 mL after pre-operative chemotherapy [2,37]. Consequently, tumor volume ≥500 mL at surgery is now a treatment stratification factor in these subsets of intermediate-risk tumors [2]. Tumor volume after pre-operative chemotherapy also predicted tumor recurrence in local blastemal-type WT [83]. Tumors with *WT1* mutations do not show volume reduction after chemotherapy, which is thought to be due to chemotherapy-induced skeletal muscle cell differentiation rather than apoptotic cell death [90,91]. However, as we will discuss in more detail in Section 7, germline and somatic *WT1* mutations are not independently associated with an increased risk of tumor recurrence. Tumor volume at diagnosis was not associated with recurrence in the SIOP 93-01 and 2001 studies [23,83].

Nephrectomy specimen weight may be a surrogate marker of tumor volume. Combined analysis of NWTS-1, 2 and 3 confirmed earlier reports that tumor weight (<550 g vs. ≥550 g) was of prognostic value in patients <2 years of age with stage I, FHWT [92,93]. These patients with a tumor weight of <550 g are now known as VLRWT patients. The prognostic significance of the 550 g cut-off could not be confirmed in UKW2 and 3, although numbers were low [26].

## 7. The Prognostic Significance of Genetic Aberrations

### 7.1. Germline Mutations

Up to 15% of WT patients present with a WT predisposition syndrome, a malformation syndrome, or germline (epi)mutation [84,94,95]. We need to acknowledge that in order to verify whether these genetic conditions have a prognostic impact on recurrence, available studies should clearly exclude contralateral metachronous tumors, since this is a known event in the setting of predisposing genetic conditions. Considering the prevalence of these predisposing genetic conditions, Dumoucel et al. identified 43 malformation and predisposition syndromes, associated with an >5% risk of developing WT, among 295 WT patients (14.6%). In fourteen patients (4.7%), the syndrome could be genetically corroborated [96]. In addition, Merks et al. observed a syndrome in 23 out of 136 WT cases (16.9%), 16 of which were genetically proven (11.8%) [97]. Finally, COG’s TARGET initiative identified germline mutations in approximately 10% of WT cases [98]. The relation between predisposing genetic conditions and tumor recurrence has been studied in a number of cases. The GPOH identified predisposition syndromes, including the presence of malformations, as an independent risk factor for progressive disease, including local and metastatic recurrence, i.e., non-metachronous disease, in stage V patients [73]. The most common predisposition syndromes and their underlying genetic defect include WAGR syndrome (Wilms tumor, aniridia, genitourinary anomalies, and range of developmental delays) (*WT1* deletion), Denys-Drash syndrome (*WT1* mutation) and Beckwith-Wiedemann Syndrome (LOH or loss of imprinting (LOI) of 11p15). Excluding metachronous tumors, both COG and SIOP studies did not observe a higher relapse rate in WAGR patients than in non-syndromic WT patients [99,100]. Similarly, excluding contralateral metachronous recurrences, RFS rates adjusted for tumor stage and histology in patients with Beckwith-Wiedemann Syndrome were similar to non-syndromic patients [101]. Germline mutations that are not associated with syndromic WT include mutations in *ARID1A*, *EP300*, *CHEK2*, *REST* and *PALB2* [84]. The latter two mutations may be associated with familial WT, and mutated *PALB2* was additionally found to be associated with DA [98,102]. Larger cohorts must be studied to determine the absolute relapse rates associated with these non-syndromic germline mutations. Finally, germline mutations in *TRIM28* have been found to predispose to familial and non-familial WT [103,104]. WTs with *TRIM28* mutations were primarily associated with epithelial histology and patients, both treated according to SIOP and COG guidelines, showed excellent RFS rates (e.g., Hol et al. reviewed all previously reported cases of *TRIM28* mutations in WT (including germline and somatic mutations), and none of the 13 patients for which follow-up data was available relapsed) [104,105,106]. *TRIM28* mutations may thus have prognostic value. Whether the prognostic value persists independently of epithelial histology remains to be determined [107,108].

### 7.2. Somatic Mutations

The review by Treger et al. provides a comprehensive overview of common somatic mutations in WTs and their clinical relevance [84]. The most relevant are mutations in *MYCN* and *TP53*, as previously described in Section 6.1 Anaplasia, as well as *WT1*, *IGF2* (located at chromosome region 11p15), *CTNNB1*, *WTX*, *SIX1/SIX2* and microRNA processing genes (miRNAPGs). Somatic *WT1* mutations are observed in 10–20% of WT cases, and, in general, do not appear to have independent prognostic value [84,109,110]. *WT1* mutations, however, were found associated with recurrence in VLRWT patients specifically. This finding could not be reproduced in the AREN0532 study. The AREN0532 did confirm that VLRWT patients with LOH or LOI of 11p15 were more likely to experience tumor recurrence than patients with retention of imprinting [32,111]. In a cohort of 23 WTs, not limited to VLRWT, Coorsens et al. observed that hypermethylation of *H19* (thought to result from LOI of 11p15 and leading to *IGF2* overexpression [112,113]) was directly associated with clonal nephrogenesis and subsequent WT formation. The authors suggest that the extent of clonal nephrogenesis may serve as a prognostic marker for WT recurrence, although future studies must confirm this hypothesis [114]. *β*-catenin (encoded by *CTNNB1*) and WTX (encoded by *WTX)* play a key role in the Wnt signaling pathway, in which wild type β-catenin co-activates transcription, and WTX is suggested to suppress Wnt signaling [115]. Altered Wnt signaling, however, was not found to be associated with risk of WT recurrence [91,109]. Finally, concurrent mutations in both *SIX1* or *SIX2* and a miRNAPG (including *DROSHA* or *DGCR8*) resulted in a decrease in EFS in a study by the COG [116]. This association was not found in tumors treated with pre-operative chemotherapy, but it was shown that *SIX1/SIX2* and miRNAPG mutations were associated with blastemal-type WT, which is an adverse prognostic factor for recurrence [86,117]. The analysis of paired primary and recurrent tumors by AIEOP suggested that *SIX1* and miRNAPG mutations may be involved in driving tumor recurrence, suggesting potential prognostic value that must be confirmed in future studies [118,119]. Rather than adhering to a single-sample strategy, AIEOP sampled multiple sections of the same tumors to avoid missing mutations that may be heterogeneously present. Altogether, though, there are currently no known independent prognostic mutational factors for tumor recurrence.

### 7.3. Coopy Number Variations

#### 7.3.1. Gain of 1q

Gain of chromosome arm 1q is an independent prognostic marker for recurrence in WT patients, both in NWTSG/COG and SIOP-treated patients [27,28,29,47]. This is found in roughly 25% of WTs and can predict 40% of relapses [120]. The study by Gratias et al. suggested that tumors with 1q gain are more aggressive and have metastatic potential, since the prevalence of 1q gain was highest among high-stage tumors [29]. COG is planning on using 1q gain in risk stratification in the next series of studies. Future SIOP studies aim to further validate the use of 1q gain in stratification of patients who have received pre-operative chemotherapy.

#### 7.3.2. LOH of 1p and 16q

The NWTS-5 study prospectively analyzed LOH of 1p only (LOH 1p) and 16q only (LOH 16q), as well as combined LOH of 1p and 16q (LOH 1p/16q) in WT patients [21,29]. The first analysis confirmed the adverse prognostic effect of LOH 1p and LOH 16q, as well as LOH 1p/16q in patients with stage I/II FH. Considering patients with stage III/IV, only LOH 1p/16q was associated with poorer RFS. Finally, when including all stage I–IV WTs stratified by stage and age at diagnosis, only LOH 1p/16q remained significantly associated with relapse [21]. However, as discussed in Section 5.2.1, the AREN0532 study demonstrated that the combination of LOH 1p or LOH 16q in addition to lymph node positivity was associated with poorer EFS (based on log rank analysis) in patients with stage III FHWT tumors [34]. AIEOP also observed prognostic significance of LOH 1p in non-anaplastic histology tumors, yet LOH 16q and LOH1p/16q were not associated with a worse disease-free survival [15]. An analysis of patients with non-anaplastic WT registered on the UKW1–3 revealed prognostic significance of LOH 16q and of LOH 1p/16q, regardless of treatment approach [46]. However, the number of patients with LOH1p/16q in this study was rather low (*n* = 11). The prognostic effect of neither LOH 1p nor LOH 16q could be reproduced in adjusted analyses of SIOP, one of which also included UKW2-3 patients [27,47].

An updated analysis of NWTS-5 patients with longer follow-up showed that LOH 1p and LOH 16q, as well as LOH 1p/16q were associated with EFS in univariable survival analysis. When adjusted for 1q gain in multivariable analysis, the prognostic significance of LOH of 1p and/or 16q was lost, whereas the significance of 1q gain remained. However, LOH 1p and LOH 16q appeared significantly associated with 1q gain. When patients with and without 1q gain were analyzed separately, LOH of 1p and/or 16q was of independent prognostic value specifically in patients without 1q gain [29]. The findings suggest that LOH 1p and/or 16q may aid in the risk stratification of 1q-gain-negative patients. However, irrespective of 1q status, LOH 1p/16q is able to predict only 9% of recurrences [120]. Effective use of LOH 1p/16q in clinical practice is therefore limited to small patient subsets. AREN0532 or AREN0533 have used LOH of 16q/1p as treatment stratification factor, and these studies, which included therapy intensification for patients with LOH 16q/1p, showed significantly better 4-year EFS rates compared to the preceding NWTS-5 study (Table 2) [42]. The prognostic significance of LOH of 1p and/or 16q will be further validated for SIOP patients in UMBRELLA [2].

## 8. Is Prognosis Influenced by Administered Treatment?

Treatment of WT is multimodal, and the prognostic effect of treatment-related factors on patient outcome should be carefully deliberated. Rationally, inadequate treatment leads to worse prognosis, as we will further discuss in the context of resource constraints in low- and lower-middle-income countries. Nonetheless, therapeutic considerations in high-income countries may also potentially influence prognosis. The factors that will be discussed here include the two upfront treatment approaches, chemotherapeutic regimens, radiotherapy, surgical elements focused on lymph node sampling, the performance of NSS, minimally invasive surgery (MIS), and the importance of centralized expertise through international collaboration.

### 8.1. Pre-Operative Chemotherapy Versus Immediate Nephrectomy

In a randomized controlled trial, pre-operative chemotherapy, when compared to immediate nephrectomy, resulted in a reduction of tumor volume, which tended to reduce the risk of gross tumor spill, and a more favorable stage distribution among WT patients [12,121,122]. The randomized trial was intended to include a total of 350 patients. However, due to slow accrual, 205 patients were eventually randomized. Nonetheless, the data suggested that the randomized patients were representative of all patients treated for WT during the lifetime of the trial [12]. In balance to the advantages of pre-operative surgery, upfront nephrectomy permitted the option for patients who had very low risk disease and benign renal tumors to avoid chemotherapy altogether, and more accurate tissue diagnosis earlier on. Overall, EFS and OS rates were similar when comparing the two treatment strategies [1,12,123].

### 8.2. Chemotherapy: Doxorubicin

Currently, SIOP treats roughly two–thirds of all patients with VA only. The other third is prescribed additional doxorubicin in case of stage II/III tumors with intermediate-risk non-stromal or non-epithelial histology and a tumor volume of ≥500 mL after pre-operative chemotherapy, stage IV tumors and tumors with HR histology [2,124]. Doxorubicin was found to be expendable for many stage II and III IR patients treated in SIOP 2001, without affecting recurrence rates, based on a randomized controlled trial [37]. Omission of doxorubicin in stage III FH patients treated by the NWTS resulted in an increase in tumor recurrence [125]. Optimal patient selection for those needing, or not needing, doxorubicin for optimal outcomes remains an area of active investigation. Moreover, to reduce anthracycline associated cardiotoxicity, the UMBRELLA protocol aims to reduce the cumulative dose of doxorubicin in the treatment of selected metastatic WT patients, dependent on histology of the primary tumor and metastases, as well as response to pre-operative chemotherapy and surgery [2]. This reduction is based on preliminary data from the COG-initiated AREN0533 study, which suggested that a cumulative dose of 150 mg/m^2^ for patients with FH (rather than 300 mg/m^2^ prescribed during the SIOP 2001) did not negatively affect relapse-free and overall survival [1,64].

### 8.3. Radiotherapy

The current COG and SIOP guidelines for radiotherapy treatment are presented in Table S3. Various studies have investigated the possibility of reducing radiotherapy exposure to diminish radiation toxicity and studied the effect of reduced radiotherapy on tumor recurrence. Firstly, when comparing the outcome of stage I FA and DA patients treated with radiotherapy and doxorubicin on the AREN0321 study to historic controls of NWTS-1-5, lack of radiation in NWTS-1-5 was not associated with an inferior 4-year EFS [44,126]. The addition of doxorubicin did lead to an increase in 4-year EFS when compared to NWTS-5 (100% compared to 70.0%), and in the combined analysis of AREN0321 and NWTS1-5, local relapses (*n* = 3/112) were seen only in patients with DA WT [126]. SIOP stage I DA WT patients who did not receive radiotherapy had a local recurrence rate of 17%, but salvage therapy adequately preserved OS rates [127]. Hence, use of radiotherapy is not advised for treatment of SIOP stage I anaplastic (FA or DA) tumors. Secondly, Fajardo et al. reported no increased risk of recurrence when boost irradiation, in addition to flank irradiation, was omitted in IR stage III patients with positive lymph nodes [128]. Thirdly, omission of radiotherapy in patients with complete lung metastasis response after 6 weeks of pre-operative chemotherapy was shown to be safe, both in terms of OS and EFS LR and IR stage IV patients treated by the SIOP [60]. The COG recently confirmed the efficacy of this approach for FHWT in the AREN0533 trial [64]. More intense treatment with radiotherapy and chemotherapy appeared to sufficiently prevent recurrence in case of incomplete response (SIOP and COG) and in case of high risk histology (SIOP) and high risk biology (LOH 1p/16q) (COG) [60,64]. Finally, to further diminish (long-term) toxicity of radiation therapy, the UMBRELLA guideline on flank irradiation by two opposing photon beams will be adapted into a guideline for highly conformal flank target-volume delineation to diminish radiation to healthy tissues. The prognostic value of the use of, as well as the non-inferiority of the promising highly conformal flank target-volume delineation strategies is yet to be determined [129]. COG study AREN1921 incorporates such Intensity Modulated RadioTherapy (IMRT)-guided cardiac and liver sparing approaches (https://clinicaltrials.gov/ct2/show/NCT04322318 (accessed on 21 June 2021)).

### 8.4. Surgery

#### NSS and MIS

In case of unilateral disease, the standard approach to surgery of WTs is total nephrectomy during open surgery. However nephrectomy in combination with radiation therapy and use of nephrotoxic chemotherapy can lead to loss of renal function [130]. The risk of end-stage renal disease is especially high in syndromic WT (cumulative 20-year risk of 43.3% in WAGR patients, compared to 1.7% in non-syndromic patients) and in bilateral WT (cumulative 20-year risk of 3.1%, compared to 0.7% in unilateral WT (Hazard Ratio: 5.9 (95% CI: 3.2–10.6), *p*-value: <0.001)) [130,131]. Therefore, NSS on carefully selected unilateral tumors may be considered both in SIOP and COG protocols, and is strongly recommended in case of bilateral WT and predisposing syndromes [1,2]. Lymph node status is an important factor to take into account when considering NSS since patients with positive lymph nodes require radiotherapy which would jeopardize the function of the kidney initially spared by NSS in these patients. Factors that predict lymph node positivity, however, remain unidentified [132]. Strict conditions are also warranted because NSS may be associated with risk of positive resection margins or tumor spill, and subsequent tumor recurrence. In the SIOP 2001 cohort, tumor recurrence occurred in 4/91 patients (4%) after NSS for unilateral WT and 5-year EFS was similar between patients treated with NSS and total nephrectomy. However, patients treated with NSS had a smaller tumor volume at diagnosis and surgery, and a lower abdominal stage following surgery [133]. In addition to NSS, SIOP allows total nephrectomy by means of MIS in a very select group of patients. In a recent study including 50 laparoscopic nephrectomies in unilateral WT, the 3-year EFS rate was 94%, with a 4% risk of local recurrence [134]. These findings are in line with earlier reports of one local recurrence among 24 unilateral WT patients treated with MIS during the SIOP 2001 [135]. However, MIS may have the disadvantage of limiting proper local staging by the pathologist if the tumor is disrupted during removal, which is considered a surgical complication. Altogether, NSS and MIS appear not to be associated with an increased risk of recurrence, if performed after careful patient selection and by experienced surgeons. Combined minimal invasive NSS, however, is not recommended. Simultaneous introduction of two new, not fully explored factors creates a risk of failure that cannot be justified. If feasible, NSS has priority over MIS.

### 8.5. Refractory Disease during First-Line Treatment

Therapy resistance is an important cause of failure of initial treatment, especially in patients who present with very-high risk relapse [16]. However, the independent prognostic significance of refractory disease during primary treatment has not been studied in detail. Ora et al. did report on progression of local disease during pre-operative chemotherapy, which, although rare, was found to be associated with poor EFS when compared to survival of patients with stable disease in the SIOP 93-01 study when adjusted for age (in months), tumor stage and histological risk group [57].

### 8.6. Centralization of Expertise

Childhood cancer patients are more likely to survive when treated at high, compared to low volume centers, as high volume centers are more experienced in the treatment of (rare) childhood cancers, and as a result, can provide more (Wilms) tumor specific expertise [136,137,138]. The COG learned that patients with WT were more frequently treated with chemotherapy in centers with a COG membership than in non-COG centers. In these non-COG centers, 40% of patients failed to receive chemotherapy for WT. Use of chemotherapy was associated with better survival rates, irrespective of center volume [139]. During the SIOP 93-01/GPOH study, less experienced surgeons or urologists more often encountered tumor rupture during surgery, requiring tumor upstaging to stage III and postoperative treatment intensification to prevent recurrence [140]. Centralization of expertise in the form of virtual international tumor boards with participation of expert centers will aid in the equal treatment of primarily complex cases such as bilateral and DA WT, and implementation of modified highly conformal radiotherapy techniques [2,141]. These international collaborations have been taken forward by the European Expert Paediatric Oncology Reference Network for Diagnostics and Treatment (ExPO-r-Net), resulting in the European Reference Network–Paediatric Cancer (ERN PaedCan) that aims to unite specialists across Europe and provide cross-border healthcare (https://paedcan.ern-net.eu/, accessed on 21 June 2021).

## 9. The Prognostic Significance of Geographic and Socioeconomic Factors

An extensive discussion of all geographic and socioeconomic factors is beyond the scope of this review. However, whereas survival in high-income countries is excellent, outcomes of patients in low- and lower-middle-income countries leave much to be desired [142]. Therefore, the studies reported here provide a highlight of the important prognostic factors to consider in low-income countries. The French African Pediatric Oncology Group (GFAOP) reported on a 2-year EFS of 46.7% or LR and IR patients with WT in sub-Saharan Africa [143]. Improved protocol compliance resulted in a 3-year EFS of 69% for these patients, and tumors relapsed in 8.8% of cases [144]. From 2014 onwards, the SIOP PODC (Pediatric Oncology in Developing Countries) has implemented the collaborative Africa project. The five participating sub-Saharan centers combined reported a 2-year EFS of 49.9% (95% CI: 46.1–53.7%) and a relapse rate of 21% (including HR patients) [145]. Both the GFAOP and SIOP PODC encountered similar issues, most notably: limited access to health care, erratic access to radiotherapy and actinomycin-D, abandonment of treatment, death during treatment as a result of late presentation with advanced disease and treatment related toxicity due to malnutrition, and limited supportive care. Since the most common cause of treatment failure in developing countries is treatment abandonment, it is very likely that many disease recurrences remained unreported due to loss of follow-up [146].

## 10. Conclusions

Current negative prognostic factors used for patient stratification are present in only one third of WT patients who subsequently relapse. To potentially improve relapse prediction, we have provided an overview of the prognostic value of currently applied and novel factors. Tumor stage and histological subtype clearly remain relevant prognosticators, notwithstanding that the current staging system may deserve adjustments (i.e., in the differentiation between stages I and II, or in the classification of the heterogeneous stage III). Since both tumor stage definition and histologic classification are easily achievable in settings with limited resources, they remain valid prognostic classifiers in low- and middle-income countries. Patient age at diagnosis and biological markers such as 1q gain and LOH 1p/16q represent additional prognostic factors to validate or use in prospective larger experiences. It is noteworthy that access to multidisciplinary care and expertise also seem to significantly influence EFS rates of children with WT. Finally, international harmonization of definitions, diagnosis, treatment and radiology and pathology review in combination with identification of novel adverse genetic signatures may provide the opportunity to further develop the array of prognostic factors for WT recurrence.

## Figures and Tables

**Table 1:** Recognized factors with prognostic significance for treatment stratification.

COG	SIOP
Tumor stage	Tumor stage (before and after pre-operative chemotherapy)
Tumor histology	Tumor histology
Tumor weight after upfront nephrectomy	Tumor volume after pre-operative chemotherapy
Patient age	Lung nodule response (stage IV)
Lung nodule response (stage IV)	
LOH of chromosome arm 1p and 16q	
Gain of chromosome arm 1q ^1^	
LOH of chromosome arm 11p15 (in VLRWT only) ^1^	

^1^ Validated, but not yet used in prospective risk stratification in published COG trials. Abbreviations: LOH, Loss of heterozygosity; VLRWT, Very low risk Wilms tumor (i.e., age at diagnosis < 2 years, tumor stage I, non-anaplastic histology and tumor weight < 550 g).

## Supplementary Materials

The following are available online at https://www.mdpi.com/article/10.3390/cancers13133142/s1, Table S1: Current Staging system of the COG and SIOP, adapted from Phelps et al. and Fajardo et al. [17,18], Table S2: Histological classification system of the COG and SIOP, Table S3: Current radiotherapy guidelines of the COG and SIOP [1,2,5].

## Abbreviations

AH	Anaplastic histology
AHWT	Anaplastic histology Wilms tumor
AIEOP	Associazione Italiana Ematologia Oncologia Pediatrica
COG-RTC	Children’s Oncology Group Renal Tumor Committee
CT	Computed tomography
Cy	Cyclophosphamide
DA	Diffuse anaplasia
Dox	Doxorubicin
EFS	Event-free survival
ERN PaedCan	European Reference Network–Paediatric Cancer
ExPO-r-Net	European Expert Paediatric Oncology Reference Network for Diagnostics and Treatment
FA	Focal anaplasia
FHWT	Favorable histology Wilms tumor
GFAOP	French African Pediatric Oncology Group
GPOH	Gesellschaft für pädiatrische Onkologie und Hämatologie
HR	High risk
IMRT	Intensity Modulated RadioTherapy
IR	Intermediate risk
JWiTS	Japan Wilms Tumor Study
LOH	Loss of heterozygosity
LOI	Loss of imprinting
LR	Low risk
miRNAPGs	microRNA processing genes
MIS	Minimally invasive surgery
MVA	Multivariable analysis
N	Number of patients
NA	Not applicable
n.s.	Not significant
NSS	Nephron-sparing surgery
NWTSG	National Wilms Tumor Study Group
OS	Overall survival
PODC	Pediatric Oncology in Developing Countries
Ref	Reference
RFS	Recurrence-free survival
SIOP-RTSG	International Society of Pediatric Oncology Renal Tumor Study Group
UKCCLG	United Kingdom Children’s Cancer and Leukaemia Group
UVA	Univariable analysis
VA	Vincristin, Actinomycin-D
VAD	Vincristin, Actinomycin-D and Doxorubicin
VLRWT	Very low risk Wilms tumor
WAGR	Wilms tumor, aniridia, genitourinary anomalies, and range of developmental delays
WT	Wilms tumor

## Appendix A. Search Strategy

The following terms and variations thereof were used to identify relevant articles: “Wilms Tumor”, “Recurrence”, “Prognostic factor”, “Age factor”, “Sex factor”, “Race factor”, “Histology”, “Tumor volume”, “Lymph nodes”, “Residual disease”, “Tumor rupture”, “Vascular extension”, “Biopsy”, “Pre-operative chemotherapy”, “Immediate nephrectomy”, “Physician/Surgeon volume”, “Genetic aberrations”, “Developing country”. Subsequently, the search was expanded by adding the terms “SIOP”, “NWTS”, “COG”, “JWiTS”, “AIEOP” and “UKCCLG” to the search while omitting the term “Recurrence”.
