# Supplementary material for: Prognostic Factors for Wilms Tumor Recurrence: A Review of the Literature
Source: Cancers (Basel). 2021 Jun 23;13(13):3142. doi: 10.3390/cancers13133142 (PMC8268923; doi:10.3390/cancers13133142)
Supplement: Supplementary file 1 [file cancers-13-03142-s001.zip › cancers-1214658-supplementary.pdf]

cancers

MDPI

*Review*

# Prognostic factors for Wilms tumor recurrence: a review of the literature

## Supplementary Materials

**Table S1.** Current Staging system of the COG and SIOP, adapted from Phelps *et al* and Fajardo *et al* [17,18].

| Stage | COG: pre-chemotherapy | SIOP: post-chemotherapy |
|---|---|---|
| I | Confined to kidney | Tumor limited to kidney or surrounded with fibrous pseudocapsule if outside the normal contours of the kidney, the renal capsule or pseudocapsule may be infiltrated with the tumor, but it does not reach the outer surface, and it is completely resected (resection margins 'clear') |
| | Complete excision with renal capsule intact and negative resection margins | The tumor may be protruding (bulging) into the pelvic system and 'dipping' into the ureter, but it is not infiltrating their walls |
| | Lymph nodes negative for Wilms tumor spread | The vessels of the renal sinus are not involved, but intrarenal vessel involvement may be present |
| | | Fine needle aspiration or percutaneous core needle biopsy ('tru-cut') do not upstage the tumor. The presence of necrotic tumor or chemotherapyinduced changes in the renal sinus/hilus fat and/or outside of the kidney should not be regarded as a reason for upstaging a tumor. |
| II | Regional extension beyond kidney capsule, but confined to flank | The tumor extends beyond kidney or penetrates through the renal capsule and/or fibrous pseudocapsule into perirenal fat but is completely resected (resection margins 'clear') |
| | May include:<br>Tumor penetration through capsule but confined to Gerota's fascia | The tumor infiltrates the renal sinus and/or invades blood and lymphatic vessels outside the renal parenchyma but it is completely resected |
| | Infiltration into renal vein<br>Complete excision with negative resection margins<br>Lymph nodes negative for Wilms tumor spread | The tumor infiltrates adjacent organs or vena cava but is completely resected |
| III | Residual tumor, but confined to abdomen | Incomplete excision of the tumor which extends beyond resection margins (gross or microscopic tumor remains postoperatively) |
| | May include:<br>Regional lymph node involvement | Any abdominal lymph nodes are involved |
| | Peritoneal contamination by: biopsy, pre- or intraoperative tumor rupture, tumor growth through peritoneal surface or positive resection margins | Tumor rupture before or intraoperatively, if confirmed by microscopic examination |
| | | The tumor has penetrated through the peritoneal surface<br>Tumor implants are found on the peritoneal surface<br>The tumor thrombi present at resection margins of vessels or ureter, transsected or removed piecemeal by surgeon<br>The tumor has been surgically biopsied (wedge biopsy) prior to preoperative chemotherapy or surgery<br>The presence of necrotic tumor or chemotherapy-induced changes in a lymph node or at the resection margins should be regarded as stage III |
| IV | Distant metastases | Hematogenous metastases or lymph node metastases outside the abdomino-pelvic region |

**Table S2.** Histological classification system of the COG and SIOP.

| **COG:** Histological assessment pre- chemotherapy | |
|---|---|
| *Histological subtype* | *Criteria* |
| Favorable histology | No anaplasia |
| Unfavorable histology | Focal anaplasia |
| | Diffuse anaplasia |
| **SIOP:** Histological assessment post-chemotherapy | |

| *Risk group* | *Histological subtype* | *Chemotherapy-induced change* | *Histological features (% of viable tumor)* | | |
|---|---|---|---|---|---|
| | | | *Blastema* | *Epithelium* | *Stroma* |
| Low risk | Completely necrotic | 100 | 0 | 0 | 0 |
| Intermediate risk | Regressive | >66 | 0–100 | 0–100 | 0–100 |
| | Mixed | <66 | 0–65 | 0–65 | 0–65 |
| | Mixed | <66 | 11–65 | 0–89 | 0–89 |
| | Epithelial | <66 | 0–10 | 66–100 | 0–33 |
| | Stromal | <66 | 0–10 | 0–33 | 66–100 |
| | Focal anaplasia | One or two tumor foci with anaplasia. | | | |
| High risk | Blastemal | <66 | 66–100 | 0–33 | 0–33 |
| | Diffuse anaplasia | Non-localized or multifocal anaplasia, focal anaplasia with nuclear unrest in the rest of the tumor, or extra renal anaplasia. | | | |

**Table 3.** Current radiotherapy guidelines of the COG and SIOP [1,2,5].

| **Histology** | **Stage I** | **Stage II** | **Stage III** | **Stage IV** |
|---|---|---|---|---|
| *COG* | | | | |
| Favorable histology | - | - | + | +[1] |
| Unfavorable histology | + | + | + | + |
| *SIOP* | | | | |
| Low risk | - | - | - | +[2] |
| Intermediate risk | - | - | + | +[2] |
| High risk | - | + (DA only) | + | + |

[1] In case of LOH of 1p/16q or incomplete lung nodule response after 6 weeks of chemotherapy.
2 In case of lung nodules that are not responsive to 6 weeks of initial chemotherapy, or non-resectable metastases.
Symbols and abbreviations: +, radiotherapy is administered; -, no radiotherapy; DA, diffuse anaplasia.
